# Oxidized low density lipoprotein receptor 1 promotes lung metastases of osteosarcomas through regulating the epithelial-mesenchymal transition

**DOI:** 10.1186/s12967-019-2107-9

**Published:** 2019-11-12

**Authors:** Long Jiang, Shanshan Jiang, Wenjie Zhou, Jia Huang, Yongbin Lin, Hao Long, Qingquan Luo

**Affiliations:** 1 Shanghai Lung Cancer Center, Shanghai Chest Hospital, Shanghai Jiaotong University, Shanghai, 200030 China; 2 State Key Laboratory for Oncogenes and Related Genes, Key Laboratory of Gastroenterology and Hepatology, Ministry of Health, Division of Gastroenterology and Hepatology, Renji Hospital, School of Medicine, Shanghai Jiao Tong University, Shanghai Cancer Institute, Shanghai Institute of Digestive Disease, 145 Middle Shandong Road, Shanghai, 200001 China; 3 Sun Yat-sen University Cancer Center, State Key Laboratory of Oncology in South China, Collaborative Innovation Center for Cancer Medicine, Guangzhou, 510060 China; 4 Lung Cancer Institute of Sun Yat-sen University, Guangzhou, 510060 China; 5 Department of Thoracic Oncology, Sun Yat-sen University Cancer Center, Guangzhou, 510060 China

**Keywords:** OLR1, Lung metastasis, Osteosarcoma, EMT

## Abstract

**Background:**

Oxidized low density lipoprotein receptor 1 (OLR1), a type II membrane protein, has been identified as receptor for oxidized low-density lipoprotein. The current study firstly provided evidence that OLR1 regulated EMT and thus promoted lung metastases in osteosarcoma (OS).

**Method:**

All relevant experiments were conducted according to the manufacturer’s protocols. In vivo tumor xenograft experiments were carried out in 6- to 16-week-old mice, then maintained in our animal facility under pathogen-free conditions in accordance with the Institutional Guidelines and approval by local authorities. For the use of the clinical materials for research purposes, prior patient’s consent and approval from the Institute Research Ethics Committee were obtained. All statistical analyses were performed using IBM SPSS Statistics 22.0 for Windows.

**Result:**

Microarrays were adopted to explore the underlying epigenetic mechanisms related to metastasis. 11 genes were identified among total 26,890 differentially expressed genes. After validated in paired primary and metastatic tissues, OLR1 was selected in the current study. The expression levels of OLR1 were tested in 4 widely used cell lines. Cell proliferation, migration and invasion could be enhanced when OLR1 was overexpressed. OLR1 overexpression also triggered G1 to S + G2 phases of cell cycle. Accordingly, cell proliferations, migration and invasion would be reduced when OLR1 was silenced. OLR1-silencing blocked G1 to S + G2 phases of cell cycle. Also, OLR1 silencing effectively suppressed local tumor carcinogenesis and lung metastases in vivo. Moreover, silencing OLR1 repressed the expression of mesenchymal markers (Snail, Twist, and N-cadherin), but induced an epithelial marker (E-cadherin).

**Conclusion:**

This study indicated a novel molecular mechanism involving the role of OLR1 in lung metastases of osteosarcoma, strengthened the correlation between OLR1 and lung metastases.

## Background

Osteosarcoma, the most prevalent primary malignant bone tumor, arisen frequently in children and adolescents [1, 2]. Over the past 20 years, the outcomes of osteosarcoma patients with localized diseases have drastically improved because of the dramatic progress in the neoadjuvant and adjuvant chemotherapy regimens [3, 4]. Nevertheless, due to the strong tendency to metastasize of osteosarcoma, the mortality remained high for most osteosarcoma patients after developing metastases [5, 6]. Despite multidisciplinary treatment, the prognoses were usually associated with fatal outcomes, due to resistance of salvage therapeutic approaches [7, 8]. Since metastasis was accounted for the major cause of death and critical step of tumor progression in osteosarcoma patients, it was crucial need in these patients to identify the fundamental molecular and cellular mechanisms of metastases, a multistep process which mediated the migration and invasion of osteosarcoma cells from the primary sites to the distant sites [9, 10].

Oxidized low density lipoprotein receptor 1 (OLR1), a type II membrane protein with extracellular domain and a short cytoplasmic tail, has been identified as a lectin-like 50-kD receptor for oxidized low-density lipoprotein (ox-LDL) initially in endothelial cells, and subsequently in monocytes, platelets, cardiomyocytes, and vascular smooth muscle cells, as well as in renal, pulmonary, and neuronal tissues [11, 12]. OLR1, a member of the C-type lectin family, was consisted of four subunits: a C-type lectin-like fold, a single transmembrane subunit, a short N-terminal cytoplasmic domain, and a short ‘‘neck’’ or stalk region. OLR1 over-expression was reported to be associated with obvious upregulation of several oncogenes and significant increase in cell apoptosis, proliferation and migration [13–15]. Previous investigations have shown that increased serum ox-LDL levels were correlated with increased risks of breast, ovarian, and colon cancer. OLR1 was also proved to be significant in cancer cells growth and transformed state maintenance [16]. In addition, xenografts experiments indicated that OLR1 accounted for many reported oncogenic activities, such as transformation of epithelial cells, proliferation, migration, tumor growth and apoptosis [17].

Epithelial to mesenchymal transition (EMT), defined as the potential of losing epithelial characteristics followed by acquiring mesenchymal traits, which demonstrated as losing epithelial polarities and gaining mesenchymal phenotypes, was a well-studied essential procedure involving in tumorigeneses and metastases [18–20]. Previous evidence has indicated that the metastatic procedure in osteosarcoma exhibited EMT like states, exhibited by regulation of EMT-related transcription factors, such as TWIST-1, snail, and Smads, which are involved in the complex invasive and metastatic behavior of osteosarcoma progression [21–23].

Previously, we described that high expression of OLR1 was associated with short progression-free survival (PFS) in patients with squamous non-small cell lung cancer, and OLR1 could be applied in constructing a comprehensive predictive model involving patients with squamous NCSLC according to their PFS [24]. On the basis of this phenomenon, our hypothesis was that OLR1 was involved in osteosarcoma metastatic potential. In this study, OLR1 expression was analyzed in osteosarcoma cell lines and human samples. The hypothesis was that the metastasis of osteosarcoma might, at least in part, be due to high or present OLR1 expression. The speculation was that if this was the case, silencing the cells to express OLR1 might inhibit their progression, migration, and invasion potential and thus decrease their metastatic potential, which might open novel therapeutic perspectives. The silencing of OLR1 expression in osteosarcoma cell lines resulted in suppression of metastases. Moreover, high OLR1 expression in primary human osteosarcoma samples was associated with poor prognoses. Integrally, the present findings illustrated a novel step forward in comprehending the effect of gene OLR1 in osteosarcoma metastases and provided a potential target for targeted osteosarcoma therapy. The current study firstly provided evidence that OLR1 regulated EMT and thus promoted lung metastasis in osteosarcoma.

## Materials and methods

All relevant experiments were conducted according to the manufacturer’s protocols. Further details are provided in Additional file 1: Additional Methods.

### Survival analysis

To analyze the prognostic role of OLR1 expression in osteosarcoma, the current study used the KM plotter database to study the association of OLR1 expression with OS (http://kmplot.com/analysis/index.php?p=service&start=1). The database included the OLR1 expression and OS rates (with a 10 year follow-up) of 259 patients with osteosarcoma.

### Study approval

For the use of the clinical materials for research purposes, prior patient’s consent and approval from the Institute Research Ethics Committee were obtained. All animal studies were approved by the IACUC of Second Affiliated Hospital, Zhejiang University School of Medicine.

### Patients and specimens

Expression of OLR1 and internal control genes were evaluated by immunohistochemistry in 31 paired primary-metastatic osteosarcomas patients, and 30 patients with metastatic osteosarcomas and 30 baseline characteristics matched patients with non-metastatic osteosarcomas. Basic information of those patients and tumor samples is listed in Table 1. All patients had been enrolled by the SYSUCC between 2004 and 2013 and undergone surgical treatments. After surgical resection, tumor specimens and paired adjacent non-tumor specimens were collected and immediately stored in liquid nitrogen until use. All osteosarcoma specimens and matched non-metastatic specimens were confirmed by 2 senior pathologists (Shaoyan, Xi and Yong, Li).

**Table 1:** Clinicopathologic characteristics of patients with OS

Characteristic	Paired primary-metastatic OS (n = 31)	Metastatic OS (n = 30)	Non-metastatic OS (n = 30)
Age, years	12 (5–23)^a^	11 (5–21)^a^	14 (7–23)^a^
Gender (%)			
Male	21	20	20
Female	10	10	10
Primary tumor size (cm)	3.2 (2–20)^a^	3 (2–20)^a^	4 (2–20)^a^
Grade (%)			
1	25	24	26
2	1	1	0
3	3	3	2
4	2	2	2
Follow-up (months)			
Median	42.3	39.1	52.5
Range	2.5–114.3	2.5–98.5	13.2–114.3
Mean	62.9	46.7	70.4

^a^Median values are listed

### Statistical analysis

Data were indicated as the mean ± standard error of the mean (SEM). The Pearson χ^2^ test and Fisher’s exact test were used for categorical data, and an independent sample t-test, Mann–Whitney U test or one-way analysis of variance (ANOVA) with Bonferroni’s post hoc test was used for numerical data. All the results were considered to be statistically significant at values of P < 0.05 (*, P < 0.05; **, P < 0.005; ***, P < 0.0005).

All cell culture experiments, western blot, and flow cytometry were done at least in triplicate and repeated at least three times. Data analysis was performed using IBM SPSS Statistics 22.0 for Windows (SPSS Inc, Chicago, IL).

## Results

### Epigenetic screen for genes involving metastasis in OS

**Fig. 1:**
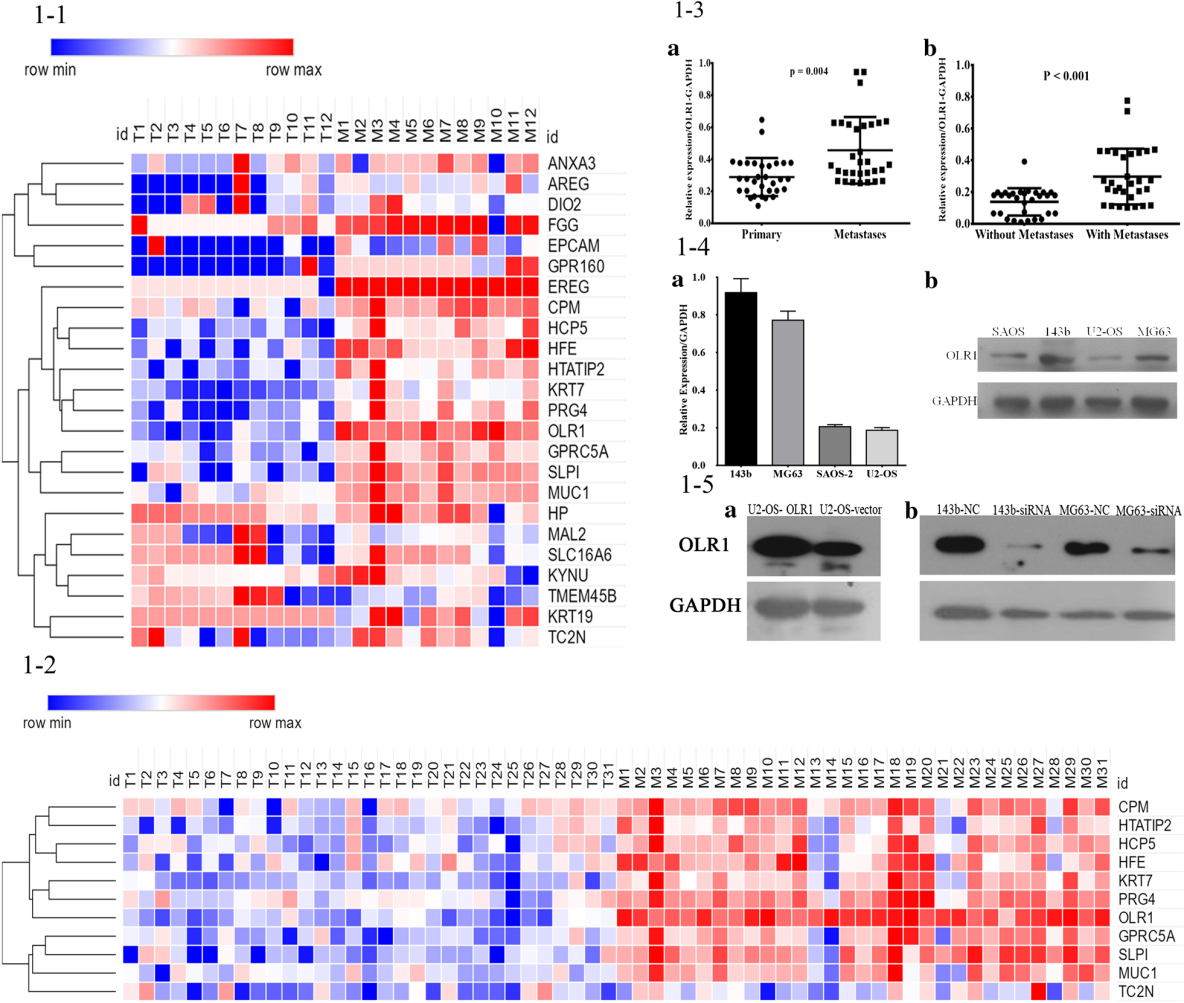
The expression levels of OLR1 in osteosarcoma. 1-1. Real-time PCR analysis to screen for activated metastasis-driving genes in osteosarcoma. Heatmap clustering of expression array data obtained from 12 pairs of primary and metastatic tumors tissues. 1-2. Real-time PCR analysis to screen for activated metastasis-driving genes in osteosarcoma. Heatmap clustering of expression array data obtained from 31 pairs of primary and metastatic tumors tissues. 1-3. The expression levels of OLR1 in osteosarcoma tissues. **a** The expression levels of OLR1 in 31 pairs of human primary osteosarcoma tissues and matched metastatic osteosarcoma tissues. The expression level of OLR1 was significantly upregulated in metastatic OS tissues in comparison to the primary OS tissues (P = 0.004). **b** The expression levels of OLR1 in 30 pairs of human primary osteosarcoma tissues from metastatic and non-metastatic osteosarcoma patients. Higher OLR1 expression level was observed in the primary osteosarcoma tissues from metastatic patients (P < 0.001). 1-4. The expressions of OLR1 in osteosarcoma cell lines. **a** It was shown by qRT-PCR that the expressions of OLR1 were low in U2-OS and SAOS-2 cell lines, but high in 143b and MG63 cell lines. **b** It was validated by Western blotting that the expressions of OLR1 were low in U2-OS and SAOS-2 cell lines, but high in 143b and MG63 cell lines. 1-5. The expressions of OLR1 in osteosarcoma cell lines after relevant treatment. **a** After the full-length OLR1 gene was overexpressed in U2-OS cells, a high expression was achieved. **b** Lentiviral constructs encoding OLR1-targeting shRNAs were transferred into MG63 and 143b cells. The expression levels of OLR1 were validated by Western blotting

To explore the underlying epigenetic mechanisms related to metastasis, microarrays were adopted to analyze 4 patients with metastatic OSs, whose primary and metastatic tumors were available. Approximately 542 genes were differentially expressed between the 4 pairs of primary and metastatic tumors tissues with a false discovery rate of 0.01 (Additional file 1: Figure S1). The subsequently statistical analysis revealed that 24 genes were upregulated in metastatic tumors when compared with primary tumors with P < 0.01 and greater than a twofold difference in expression levels among the above 542 genes (Fig. 11-1). Some of these upregulated genes, including HTATIP2, PRG4, and HFE, were previously reported to be involved in cancer metastasis [25–27]. The identification of these known metastasis-related genes suggested that our high-throughput platform was effective for the discovery of genes that drive OS metastasis.

**Fig. 2:**
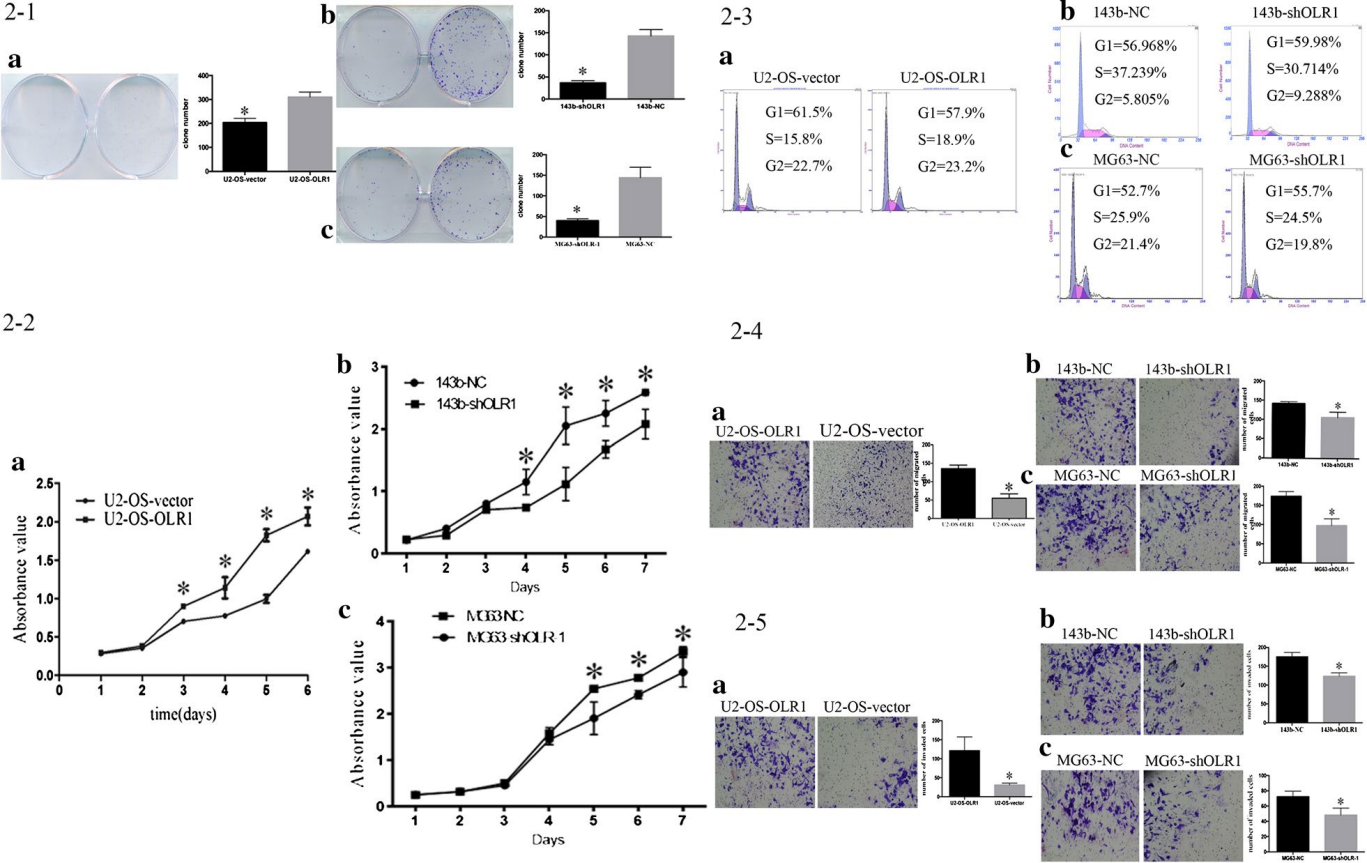
The effect of OLR1 in osteosarcoma cells. 2-1. The effect of OLR1 on colony formation assays in osteosarcoma cell lines. **a** Culturing equal numbers of OLR1-overexpressing U2-OS cells and vector controls for 48 h, much higher cell densities for the OLR1-overexpressing U2-OS cells were observed. **b** Culturing equal numbers of OLR1-silencing 143b cells and controls for 48 h, much lower cell densities for the OLR1-silencing 143b cells were observed. **c** Culturing equal numbers of OLR1-silencing MG63 cells and controls for 48 h, much lower cell densities for the OLR1-silencing MG63 cells were observed. 2-2. The effect of OLR1 on cell proliferations in osteosarcoma cell lines. **a** Cell proliferations of OLR1-overexpressing U2-OS cells were enhanced. **b** Cell proliferations of OLR1-silencing 143b cells were reduced. **c** Cell proliferations of OLR1-silencing MG63 cells were reduced. 2-3. The effect of OLR1 on cell cycle in osteosarcoma cells. **a** OLR1 overexpression triggered G1 to S + G2 phases of cell cycle in U2-OS cells. **b** OLR1-silencing blocked G1 to S + G2 phases of cell cycle in 143b cells. C: OLR1-silencing blocked G1 to S + G2 phases of cell cycle in MG63 cells. 2-4. The effect of OLR1 on migratory abilities in osteosarcoma cells. **a** The overexpression of OLR1 in U2-OS cells increased migratory abilities. **b** 143b cells migratory abilities were suppressed when OLR1 was silenced. **c** MG63 cells migratory abilities were suppressed when OLR1 was silenced. 2-5. The effect of OLR1 on invasive abilities in osteosarcoma cells. **a** The overexpression of OLR1 in U2-OS cells increased invasive abilities. **b** 143b cells invasive abilities were suppressed when OLR1 was silenced. **c** MG63 cells invasive abilities were suppressed when OLR1 was silenced

**Fig. 3:**
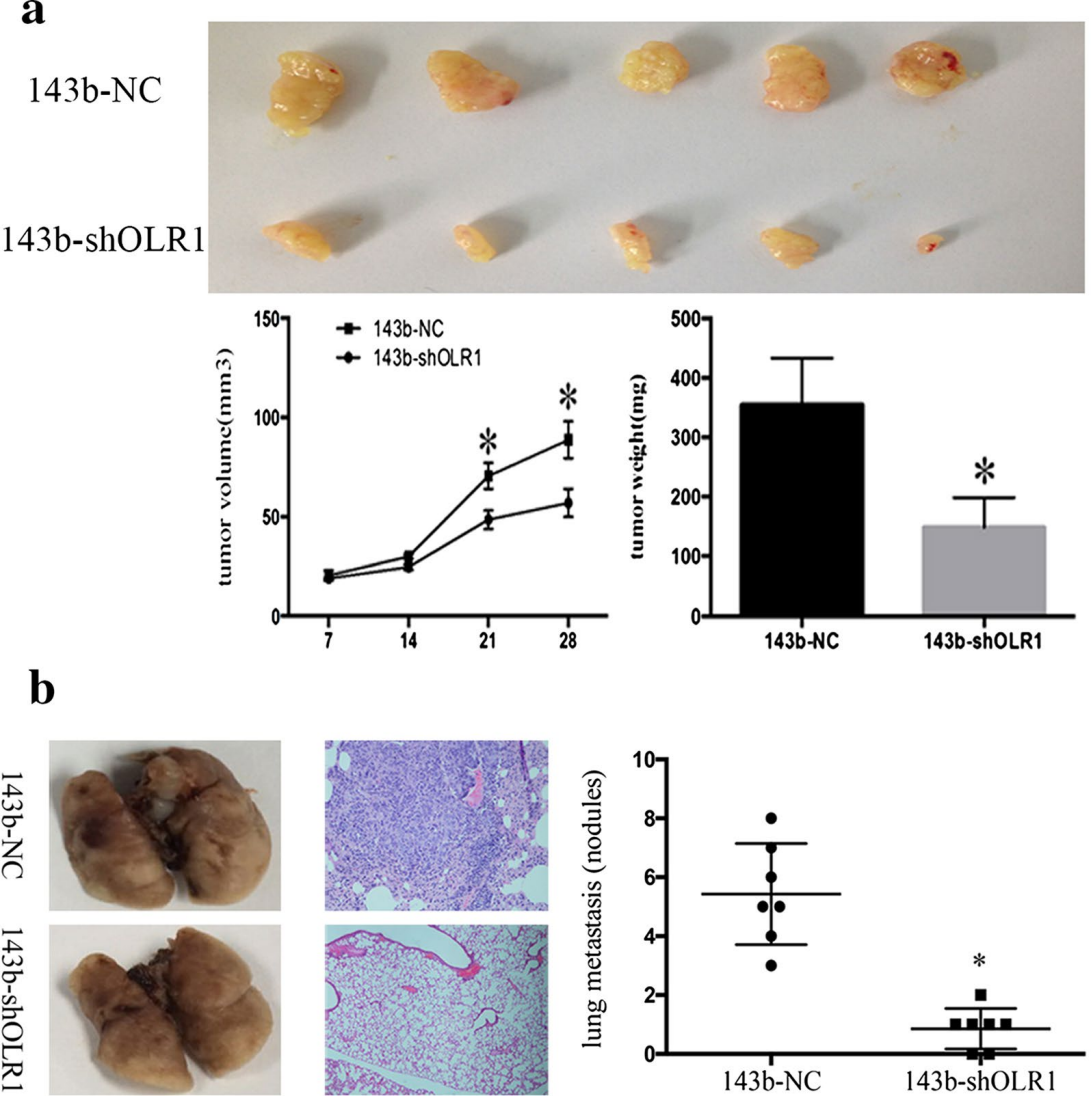
The effect of OLR1 on local tumor carcinogenesis and lung metastases in osteosarcoma cells. **a** The effect of OLR1 on local tumor carcinogenesis in osteosarcoma cells. Female BALB/c immune-deficient mice were inoculated subcutaneously with stable OLR1-silencing 143b cells and respective controls. The subcutaneous tumors were removed to make frozen specimens; along with the tumor volume and weight were measured. OLR1 silencing suppressed local tumor carcinogenesis of osteosarcoma cells in vivo. **b** The effect of OLR1 on lung metastases in osteosarcoma cells 143b-shOLR1, and its control cells were cultured and injected through the tail vein into female BALB/c immune-deficient mice. OLR1 knockdown in 143b cells caused reduction in the number of lung metastases

Subsequently, using real-time PCR, we determined the expression levels of 11 differentially expressed genes selected among the 24 genes (Fig. 11-2). Among these genes, our results demonstrated that the greatest difference in expression levels of OLR1 was observed between metastatic and primary tumors. The expression levels of OLR1 were examined in 31 pairs of human primary OS tissues and matched metastatic OS tissues. The data validated that the expression level of OLR1 was significantly upregulated in metastatic OS tissues in comparison to the primary OS tissues (P = 0.004, Fig. 11-3a). Furthermore, the expression levels of OLR1 were also examined in 30 pairs of human primary OS tissues from metastatic and non-metastatic OS patients. Similar tendency was also observed as significantly higher OLR1 expression level in the primary OS tissues from metastatic patients (P < 0.001, Fig. 11-3b). All the above findings supported the notion that OLR1 might act as a tumor promotor in OS and play a key role in inducing OS metastasis. Taken together, OLR1 was selected in the current study, while other candidate genes, such as PRG4, would be investigated in separate studies.

### Differential expression of OLR1 in OS cell lines

**Fig. 4:**
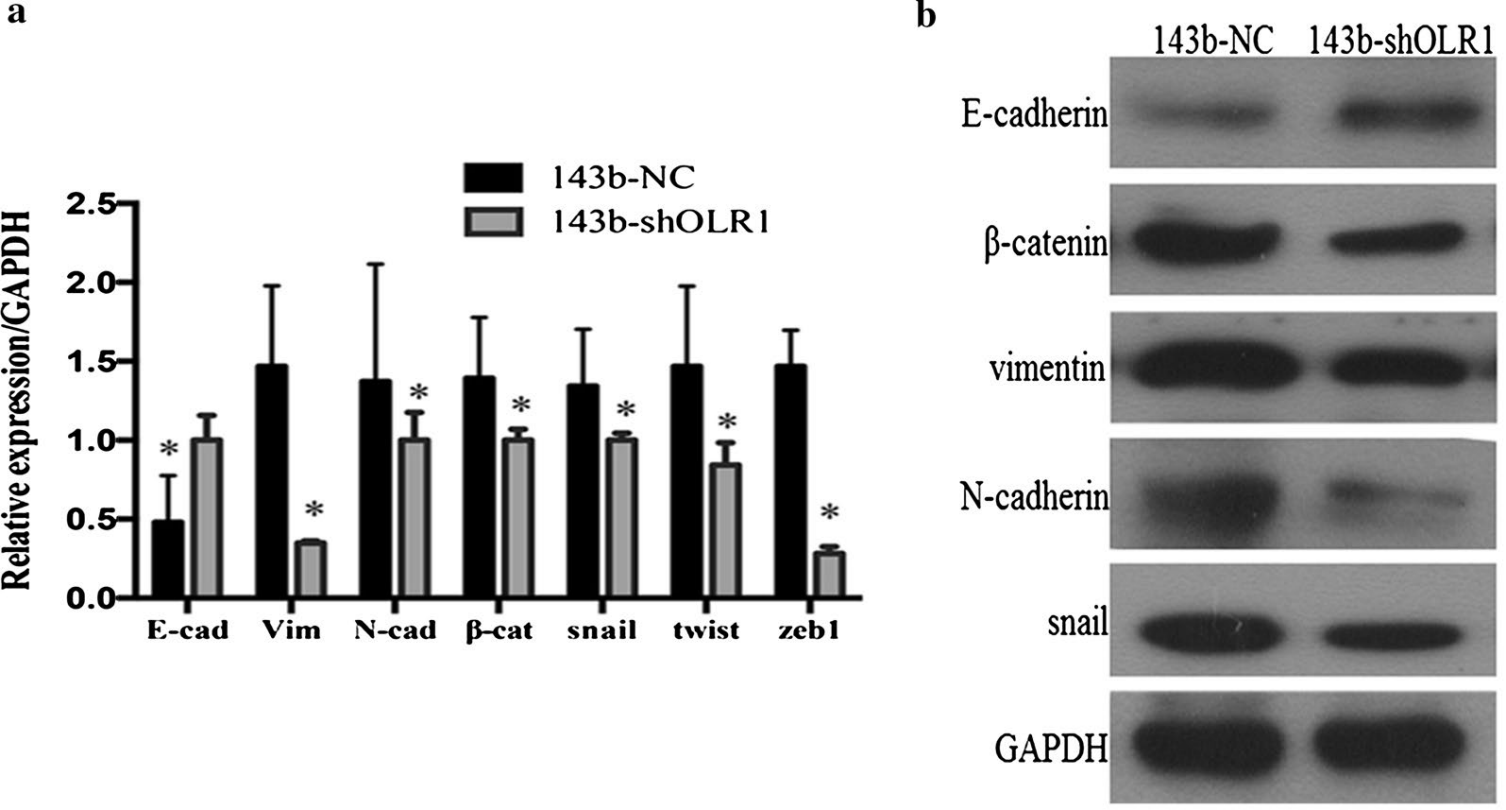
The effect of OLR1 on mesenchymal transformation in osteosarcoma cells. The epithelial and mesenchymal cell markers were exploited in stable OLR1-silencing 143b cells and respective negative controls. Silencing OLR1 repressed the expression of mesenchymal markers (Snail, Twist, and N-cadherin), but induced an epithelial marker (E-cadherin), which were confirmed by qRT-PCR (**a**), and Western blotting (**b**)

The expressions of OLR1 were investigated in four OS cell lines (U2-OS, SAOS-2, 143b, and MG63) by qRT-PCR (Fig. 11-4a) and Western blotting (Fig. 11-4b). The results indicated that the expression levels of OLR1 were low in U2-OS and SAOS-2 cell lines, but high in 143b and MG63 cell lines (Fig. 11-4a, b).

### Overexpression of the OLR1 gene in OS cells

**Fig. 5:**
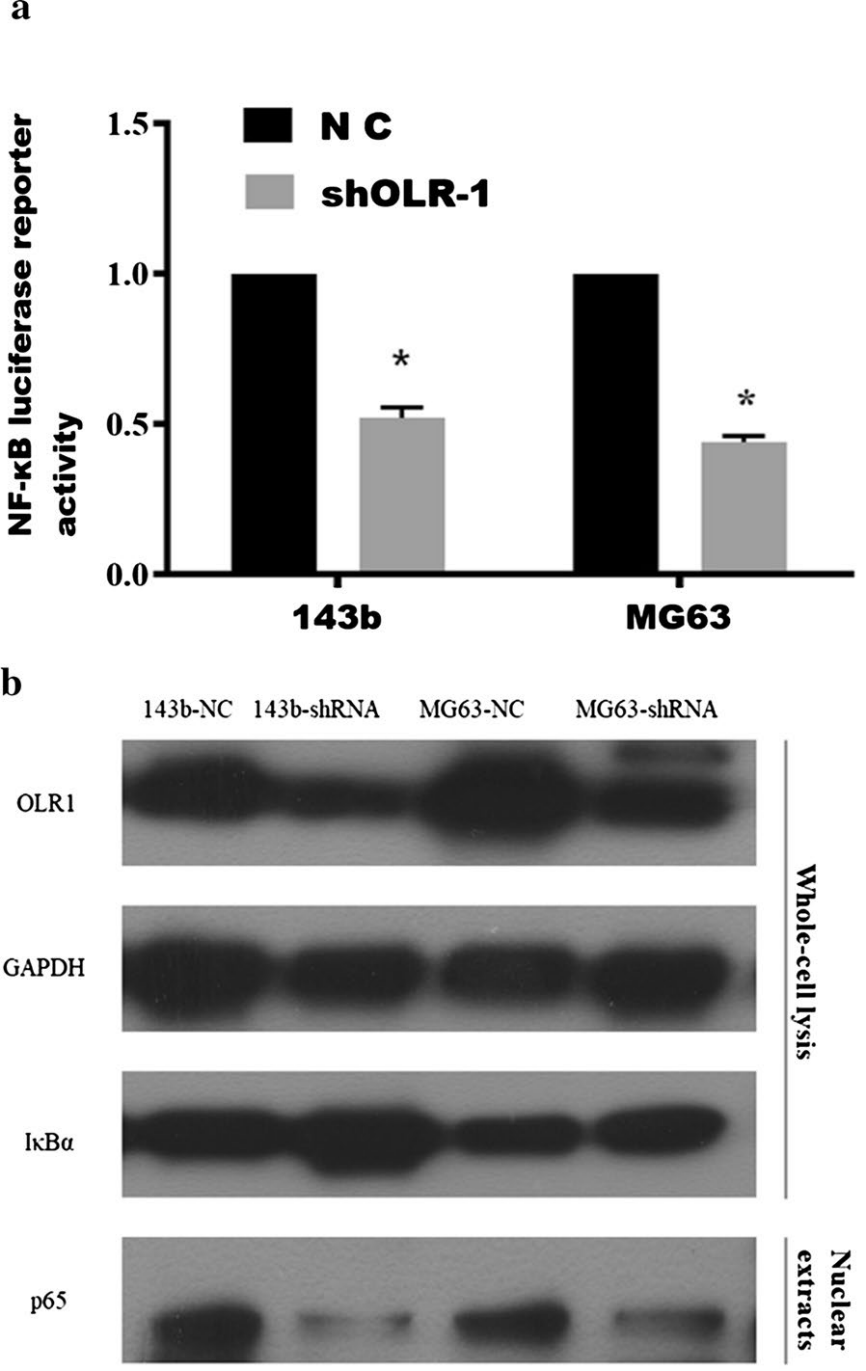
OLR1 activates NF-κB signaling pathway in OS cells. NF-κB transcriptional activity was assessed by luciferase reporter constructs in the indicated OS cells. **a**. Error bars represent the mean ± S.D. of three independent experiments. *P < 0.05. Western blotting of nuclear NF-κB/p65 expression in the indicated OS cells (**b**)

From the present background, the speculation was that overexpression of the OLR1 gene in OS cells might enhance their metastatic abilities. To test this hypothesis, the full-length OLR1 gene was overexpressed in U2-OS cells, which expressed low levels of OLR1. As shown in Fig. 11-5a, at the protein level, a high expression of the recombinant full-length OLR1 in U2-OS cell lines was achieved.

### OLR1 overexpression enhanced cell proliferation

When culturing equal numbers of OLR1-overexpressing cells and vector controls for 48 h in proper medium, much higher cell densities for the OLR1-overexpressing cells were observed by colony formation assays when compared to the vector controls (Fig. 22-1a). Consistently, cell proliferations of OLR1-overexpressing cells were found to be significantly enhanced by MTT assays (Fig. 22-2a).

### OLR1 overexpression alternated cell cycles of OS cells

Cell cycle analysis were performed to determine whether OLR1 enhanced cell proliferation via alteration of the cell cycle. As expected, OLR1 overexpression triggered G1 to S + G2 phases of cell cycle (Fig. 22-3a). The results indicate the role for OLR1 in OS cells as a cell cycle priming factor.

### OLR1 overexpression promoted the migration and invasion of OS cells in vitro

The positive effect of OLR1 on migration and invasion was determined. As expected, the ectopic overexpression of OLR1 in U2-OS cells increased their migratory (Fig. 22-4a) and invasive (Fig. 22-5a) abilities. These results demonstrated that OLR1 positively regulated OS cells migration and invasion.

### Effect of OLR1 gene silencing on tumor cell behaviors

Moreover, to explore whether silencing of OLR1 would influence cell proliferation, cell cycles, migration, and invasion, lentiviral constructs encoding OLR1-targeting shRNAs were transferred into MG63 and 143b cells. Expression of OLR1 was confirmed by Western blotting. (Figure 11-5b). As shown in Fig. 22-1b, c, when culturing equal numbers of OLR1-silencing cells and controls for 48 h in proper medium, much lower cell densities for the OLR1-silencing cells were observed by colony formation assays when compared to the controls. Cell growths of 143b and MG63 were clearly reduced. Next, cell proliferations of OLR1-silencing cells were found to be significantly reduced when measured by MTT assays (Fig. 22-2b, c).

In addition, cell cycle analyses were performed to determine whether OLR1-silencing inhibited cell proliferation via affecting cell-cycle distribution. In contrast to OLR1 overexpression, OLR1-silencing blocked G1 to S + G2 phases of cell cycle (Fig. 22-3b, c). The results added evidence of the role for OLR1 in OS cells as a cell cycle priming factor.

Furthermore, the effect of OLR1-silencing on migration and invasion was determined. As expected, OS cells migratory (Fig. 22-4b, c) and invasive (Fig. 22-5b, c) abilities were significantly suppressed when OLR1 was silenced. These results indicated the negative role of OLR1-silencing on OS cells migration and invasion.

### Silencing OLR1 inhibited the tumorigenicities and metastases of OS cells in vivo

To examine whether the silence of OLR1 inhibits tumors tumorigenicities and metastases in vivo, female BALB/c immune-deficient mice were inoculated subcutaneously with stable OLR1-silencing 143b cells and respective controls. Five mice were analyzed per treatment group. The tumor sizes were measured every week. Five weeks later, the subcutaneous tumors were removed to make frozen specimens; along with the tumor volume and weight were measured. As shown in Fig. 3a, OLR1 silencing effectively suppressed local tumor carcinogenesis of OS cells in vivo.

The lung is the most common site of OS metastasis. Therefore, xenograft mice models were applied to determine whether OLR1 shRNA reduced lung metastasis in 143b -injected mice in vivo. 143b-shOLR1, and its control cells were cultured and injected through the tail vein into female BALB/c immune-deficient mice. Eight mice were analyzed per treatment group. After 8 weeks, lung metastases were checked. OLR1 knockdown in 143b cells caused a significant reduction in the number of lung metastases (Fig. 3b). These findings confirmed that the silencing of OLR1 expression inhibited lung metastases in vivo.

### Silencing of OLR1 expression represses mesenchymal phenotype

Previous investigations have proposed that EMT was associated with cancer cell progression, invasion, migration, tumor metastases, and progression [28, 29]. Snail and Twist were proved to be transcription repressors who have a critical role in EMT both during the tumorigenicities and metastases. Of note, as a repressor of E-cadherin, Snail leads to the loss of polarity and morphologic change by repressing E-cadherin. To contribute to this phenomenon, whether the oncological function of OLR1 was regulated by the mesenchymal transformation of OS was investigated. The epithelial and mesenchymal cell markers were exploited in stable OLR1-silencing 143b cells and respective negative controls (control shRNAs). Eventually, the results confirmed by qRT-PCR (Fig. 4a), and Western blotting (Fig. 4b) indicated that silencing OLR1 repressed the expression of mesenchymal markers (Snail, Twist, and N-cadherin), but induced an epithelial marker (E-cadherin). These results revealed that silencing of OLR1 could suppress cell metastases by inhibit the mesenchymal transformation of OS.

### OLR1 activates NF-κB signaling pathway

Previous experiments considered that EMT is a common tumor metastasis mechanism, and NF-κB pathway plays an important regulatory role in the process of EMT [30]. The results showed that silencing OLR1 decreased NF-κB-dependent luciferase activity in OS cells via luciferase assay (Fig. 5a). Furthermore, downregulating OLR1 decreased nuclear translocation of NF-κB/p65 via cellular fractionation and western blotting analysis (Fig. 5b). These results indicate that OLR1 activates NF-κB signaling in OS cells.

### Prognostic value of OLR1 in human osteosarcoma patients

**Fig. 6:**
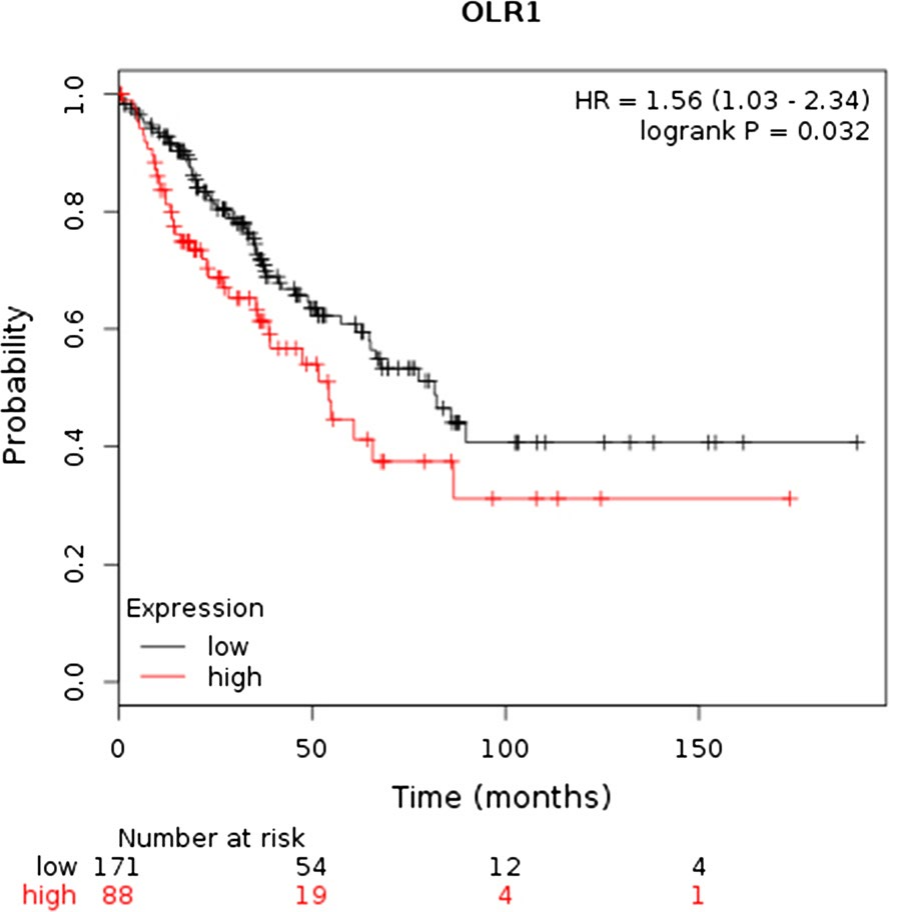
Prognostic value of OLR1 in patients with osteosarcoma. Survival curves are plotted for patients with osteosarcoma. *HR* hazard ratio

The current study used the KM plotter database and analysis tool to determine the prognostic value of OLR1 for osteosarcoma. Survival curves were generated for patients with osteosarcoma (n = 259). Compared with high expression, low expression of OLR1 was associated with higher OS for patients with osteosarcoma (Fig. 6). The HR for OLR1 was 1.56 [95% confidence interval (CI) = 1.03–2.34] and P = 0.032 (Fig. 6).

## Discussion

The approach of multidisciplinary treatment for osteosarcoma had led to improved survival [31–33]. Nevertheless, patients with metastatic diseases continued to have poor prognoses [34, 35]. Therefore, identifying the fundamental molecular and cellular mechanisms of metastases were warranted. Our previous work illustrated that high expression of OLR1 was associated with short PFS in patients with squamous non-small cell lung cancer, and OLR1 could be applied in constructing a comprehensive predictive model involving patients with squamous NCSLC according to their PFS [24]. In the current study, we show that OLR1 could promote metastases of osteosarcoma in vitro and in vivo.

Osteosarcomas, mainly occurred in adolescents and children, were high-grade malignant bone neoplasms characterized by early metastases into lungs [36, 37]. After comprehensive treatment, approximately 20% of mortalities might be attributed to lung metastases in patients with osteosarcomas [38, 39]. Therefore, identifying effective tumor-related factors to prevent osteosarcoma metastases would be critical. In this study, novel insights involved in the OLR1 function and its role in osteosarcoma metastases were gained. The OLR1 expression level was related to cell migratory potential in vitro, and its downregulation could inhibit EMT and tumor metastases in vivo. The current observation provided a potential opportunity in osteosarcoma targeted therapy involved in OLR1 gene.

Cancer metastases were complex steps including cells detaching from primary sites and forming secondary tumors at distant sites [40–42]. Regulation of cell adhesion and migration had been identified as a critical step of the metastatic process [43–45]. The complex and dynamic networks involving invasion, migration, which were mediated by numerous intracellular mediators, were significant for tumor metastases. Potential elements that could govern or control tumor cell migration and invasion would be important in the approach of metastases to distal organs [46–48].

As a potential paradigm of interpreting invasive and subsequently metastatic behavior in cancer progression, abundant attentions were attracted by EMT [49–51]. Here, we described that OLR1 induced EMT, thus subsequently promoted lung metastases in osteosarcomas.

Recent studies have indicated that OLR1 regulated the genes involved in cancer cell migration and invasion, thus played a crucial role in cancer progression. OLR1 had been implicated in the tumorigenesis or progression of several types of cancers by a series of studies [13, 16].

In the present study, we identified OLR1 as a highly-overexpressed protein in osteosarcomas that seemed critically involved in the malignant behavior of this disease, presumably, in part, via the EMT approach. Our data support a role of OLR1 overexpression for the maintenance of invasiveness and metastatic spread. Furthermore, we showed that OLR1 is also important for colony formation and proliferation of osteosarcoma cells in vitro and for tumorigenicity and metastases in vivo.

In the current study, OLR1 was identified as a higher expressed gene in metastatic OS tissues than primary OS tissues, and in primary OS tissues from metastatic than non-metastatic OS patients. The silencing of OLR1 inhibited osteosarcoma cell invasion and migration in vitro, as well as lung metastasis in mice in vivo. Furthermore, the promotive effect of OLR1 in osteosarcoma cell migration and lung metastasis was mediated by EMT. Silencing OLR1 remarkably repressed the expression of mesenchymal markers (Snail, Twist, and N-cadherin), but induced an epithelial marker (E-cadherin) expression. This mechanism had been validated in MAPK family members, such as ERK, JNK, and p38 in terms of promoting EMT [52–54].

The process of metastasis was described as detached cancer cells escaped into the blood, then spreading to distant organs [55]. Previous studies indicated that upregulation of OLR1 would promotes migration of breast cancer cells, which was the same as our current findings [56]. One of the ligands of OLR1, phosphatidylserine, would take potential responsibility of adhesion between endothelium and cancer cells, which would be the part of the reason of OLR1 promoting cancer metastasis through regulating EMT. In addition, studies showed that OLR1 was associate with E-cadherin expression mediated by MT1-MMP metalloproteinases [57]. Moreover, as proved in the current study, OLR1 was indicated to activate NF-κB pathways, which are described to promote the expression of EMT markers, snail and slug [58].

## Conclusions

This study indicated a novel molecular mechanism involving the role of OLR1 in osteosarcoma metastases, strengthened the correlation between OLR1 and osteosarcoma progression, and examined the role of OLR1 in EMT. In the future, the molecular mechanism involving OLR1-modulating EMT will be investigated.

## Supplementary information

**Additional file 1: Figure S1.** A high-throughput method to screen for activated metastasis-driving genes in osteosarcoma. Heatmap clustering of expression array data obtained from 4 pairs of primary and metastatic tumors tissues.

## Data Availability

All data generated or analyzed during this study are included in this published article and its additional files.

## Abbreviations

OLR1: oxidized low density lipoprotein receptor 1
OS: osteosarcoma
ox-LDL: oxidized low-density lipoprotein
EMT: epithelial to mesenchymal transition
PFS: progression-free survival
SEM: standard error of the mean
ANOVA: analysis of variance
CI: confidence interval
