# Supplementary material for: Oxidized low density lipoprotein receptor 1 promotes lung metastases of osteosarcomas through regulating the epithelial-mesenchymal transition
Source: J Transl Med. 2019 Nov 12;17:369. doi: 10.1186/s12967-019-2107-9 (PMC6852786; doi:10.1186/s12967-019-2107-9)
Supplement: Supplementary file 1 — Additional file 1: Figure S1. A high-throughput method to screen for activated metastasis-driving genes in osteosarcoma. Heatmap clustering of expression array data obtained from 4 pairs of primary and metastatic tumors tissues. [file 12967_2019_2107_MOESM1_ESM.docx]

**Additional file 1: Additional Methods**

Cell lines and culture conditions

The osteosarcoma cell lines, U2-OS, SAOS, 143b, and MG63, were purchased from the American Type Cell Culture Collection (Manassas, VA, USA). The cells were cultured in RPMI-1640 or Dulbecco’s Modified Eagle’s Medium (DMEM) (Both Gibco/Invitrogen, Carlsbad, CA), containing 20 mM HEPES, 10% heat-inactivated (56°C for 30 minutes) fetal bovine serum (FBS) (Gibco/Invitrogen, Carlsbad, CA), 2mM L-Glutamine (Sigma, Inc., St Louis, MO), penicillin (100 U/mL), and streptomycin (100 μg/mL, Invitrogen). Adherent monolayer cultures of cells were grown to 70 to 80% confluence and then passaged approximately every 3-5 days and maintained at 37°C in a constant humidified atmosphere with 5% CO2 sterile incubator. Cell lines were checked routinely for purity and mycoplasma contamination. To avoid losing reproducibility, cell lines would not be cultured after six passages since recovery from original frozen stocks.

RNA interference

To transiently inhibit OLR1, cells at around 70% confluence were transfected with the indicated siRNA using Lipofectamine® RNAiMAX Reagent (Invitrogen Life Technologies, Carlsbad, CA, USA) according to manufacturer’s instruction. 72 hours after transfection, cells were detached with trypsin/EDTA, suspension, and allowed to grow overnight before treatment. The siRNAs directed against OLR1 and relevant negative controls were synthesized by GenePharma (Shanghai, China). Knockdown efficiency was evaluated by Western blotting. siRNAs sequences are provided in the Supplementary Information.

Transfection with OLR1 shRNA plasmids and relevant controls

To establish the OLR1 stable knockdown OS cell lines, OLR1 shRNAs plasmids used for gene silencing were constructed by GenePharma (Shanghai, China). 143b and MG63 cells were transfected with the OLR1 shRNA plasmids and relevant controls using Lipofectamine 2000 (Invitrogen Life Technologies, Carlsbad, CA, USA) according to the manufacturer’s instructions. OLR1-shRNA and control cells were puromycin selected (10 μg/mL).

Transfection with OLR1 overexpression vectors

For overexpression of full-length OLR1, the OLR1-overexpressing recombined Lentivirus vector and the control vector were purchased by GenePharma (Shanghai, China). Lentiviral infection was performed by adding virus solution to 143b and MG63 cells in the presence of 5 μg/ml polybrene (Sigma-Aldrich, St. Louis, MO, USA). After infection for 48 h, the cells were selected in the presence of 10 μg/ml puromycin, and puromycin-resistant cells were collected and cultured.

RNA isolation and quantitative real-time polymerase chain reaction (qRT-PCR)

Total RNA from tissue samples and cell lines were extracted with the PureLink™ FFPE Total RNA Isolation Kit (Invitrogen) according to the manufacturer’s protocols. The cDNA was generated by using iScript™ cDNA Synthesis Kit (Bio-Rad Laboratories). qRTPCR analysis was performed on ABI PRISM® 7900HT Sequence Detection System (Applied Biosystems) using ABsolute™ Blue QPCR SYBR® Green ROX Mix (Thermo Scientific). All target gene and internal control primers were purchased commercially (Applied Biosystems, Foster City CA, USA), and the sequences as Supplemental Information. All assays were conducted in triplicate. The cycling conditions consisted of initially polymerase activation at 95°C for 15 min, following 45 cycles of denaturing at 95°C for 15 s, annealing at 58°C for 30 s, and extension at 72°C for 30 sec, finally extension at 72°C for 10 min. The threshold was set higher than the nontemplate control background and within the linear phase of target gene amplification for calculating the cycle number at which the transcript was detected (denoted as CT). In every experiment, non- RT- and non-template controls were run and amplification reactions were checked for non-specific products by melting curve analysis and agarose gel electrophoresis. Normalized gene expression values were calculated by DataAssist™ Software (Thermo Fisher Scientific) using the comparative CT (2-ΔCt) method, where the expression was normalized to two reference genes illustrating the best stability across samples. A list of the primers sequences used and PCR products sizes were displayed in the Supplementary Information.

Western blotting

Preparation of cell lysate and conditioned medium

After the cultured cells reached to their 70–80% confluence, the medium was removed. The cells were washed twice with PBS and lysed in 1 ml/100 mm dish of cell lysis buffer (1 % Triton X-100, 20 mM Tris–HCl pH 8.0, 137 mM NaCl, 10 % glycerol, 2 mM EDTA pH 8.0). Then the cells were harvested and lysed with RIPA lysis buffer supplemented with protease and phosphatase inhibitors (Santa Cruz Biotechnology, Inc.). Lysates were cleared by centrifugation at 12,000 × g at 4°C. Total protein concentration was measured using a bicinchoninic acid assay kit (Bio-Rad Laboratories) with bovine serum albumin as a standard. The protein was denatured by boiling at 100°C for 5 min in the presence of sample buffer (0.5 m Tris [pH 6.8], 10% glycerol, 10% SDS, 5% 2-mercaptoethanol, and 1% bromophenol). For each immunoblot, 30 μg of protein were separated by 10 % sodium dodecyl sulfate–polyacrylamide gel (SDS-PAGE) for 90 min at 100 V. The protein was then transferred onto a polyvinylidene difluoride membranes (Millipore, Billerica, MA). After transfer, the membranes were blocked overnight with 5% nonfat milk in Tris-buffered saline containing 0.5 % Tween 20 (TBST) for 30 min; and incubated for 2 hours with anti-FGF2 antibody (Abcam) at a 1:1000 dilution or with anti-β-actin antibody (Abcam) at a 1:10,000 dilution. Membranes were then washed three times with TBST and incubated for 2 h with the second antibody, a peroxidaseconjugated monoclonal anti rabbit or anti mouse antibody (Amersham) at either a 1:5000 dilution or a 1:50,000 dilution, then washed three times with TBS-T. The labeled bands were revealed by chemiluminescence using ECL Western blotting detection reagents (Amersham, Arlington Heights, IL) and exposed to Kodak XOmat film. Density of each band was quantitated with NIH Image software. All blots were reprobed with an antibody directed against GAPDH as a control for equal loading. All immunoblots were performed at least three times with samples from at least three independent transfections.

The following primary antibodies were employed: anti-OLR-1 (1:1000 dilution; Proteintech Group), anti-GAPDH (1:5000 dilution; Cell Signaling Technology), anti-E-caderin (1:1,000 dilution; Cell Signaling Technology), anti-β-catenin (1:500 dilution; Cell Signaling Technology), anti-E-caderin (1:1,000 dilution; Cell Signaling Technology), anti-Vimentinentin (1:1,000 dilution; Cell Signaling Technology), anti-N-cadherin (1:1,000 dilution; Cell Signaling Technology), anti-snail (1:500 dilution; Cell Signaling Technology), anti-twist (1:1,000 dilution; Cell Signaling Technology), anti-zeb1 (1:1,000 dilution; Cell Signaling Technology). Membranes were washed four times with TRIS-buffered saline with Tween-20 for 10 min. After washing, membranes were probed with HRP-conjugated secondary antibody and visualized using a chemiluminecent system (Cell Signaling Technology, Danvers, MA, USA). Densitometric analysis was performed using GS-800 Imaging Densitometer and Quantity One 4.6.9 software (Bio-Rad Laboratories, Hercules, CA, USA).

Immunohistochemistry

Formalin-Fixed and Parrffin-Embedded (FFPE) blocks of tissues from patients with OS were obtained from Sun Yat-sen University Cancer Center tissue banks. Resected tissues from mice were washed in saline, fixed in 10% formalin buffer, and embedded in paraffin. IHC staining was performed as the following, briefly, sections (thickness, 4 μm) were deparaffinized and rehydrated in a graded series of alcohol solutions. For antigen retrieval, slides were immersed in ethylenediamine tetra-acetic acid (EDTA; 1 mmol/L, pH8.0) and boiled for 15 min in a microwave oven. Endogenous peroxidase activity was blocked in 3% H2O2 at room temperature for 15 min, and non-specific binding was abolished by 5% bovine serum albumin (BSA) for 30 min. Sections were then stained with anti-OLR-1 (rabbit anti-galectin-3 polyclonal antibody; 1:250 dilution; Proteintech) antibody at 4°C overnight. After washing with phosphate-buffered saline (PBS), sections were incubated with horseradish peroxidase (HRP)-conjugated secondary antibody (Envision Detection kit, GK500705, Gene Tech, Shanghai, China) at room temperature for 30 min. After washing thrice with PBS, antibody complexes were colored with 3, 3’-diamino benzidine and then counterstained with hematoxylin. Slides were dehydrated and evaluated.

Flow cytometry

For DNA content, cells were fixed in ice-cold 70% ethanol at -20℃ overnight and stained with propidium iodide (50 mg/mL) plus RNase (5 mg/mL) in PBS. The blocking of nonspecific antibody binding was achieved using FACS wash buffer (2% FCS in PBS). Data acquisition was performed on a flow cytometer (Beckman Coulter), and the flow cytometry data were analyzed using Beckman Coulter (Fullerton, CA, USA) software.

Cell proliferation assay

The in vitro cell proliferation of osteosarcoma cells was measured using the MTT (3-(4,5-dimethylthiazol-2-yl)-2,5-diphenyltetrazolium bromide) method. 1×10e4 to 3×10e4 cells were seeded into 96-well plates with 200 mL media containing 10% FBS and allowed to grow up to 72 hours. In the indicated time periods, 0.1 ml of spent medium was replaced with an equal volume of fresh medium containing MTT 0.5 mg/ml. Plates were incubated at 37 °C for 4 h, and then, the medium was replaced with 0.1 ml of DMSO (Sigma), and plates were agitated at room temperature for 10 min. The absorbance was measured at 490 nm using an enzyme-labeled analyzer. Three independent experiments (three replicates in each) were performed. Gene knockdown was monitored by qRT-PCR.

Wound-healing assay

For wound-healing migration assays, the cells were seeded on 6-well plates at a density of 2 × 105 cells/ per well in a culture medium. At 24 h after seeding, the cells were treated with the indicated inhibitors or a neutralized antibodies for 30 min or transfected with shRNA plasmids for 24 h. After pretreatment, the confluent monolayer of the culture was scratched using a fine pipette tip, and incubated with recombinant OLR1 for 24 h and migration was observed using microscopy. The rate of wound closure was observed at the indicated times.

Migration assay

The migration assay was performed using the Transwell assay (Costar, NY, USA; pore size: 8 μm) in 24-well dishes with pore sizes of 8 lm (Corning, NY) until confluent. After pretreatment, approximately 1 × 10e5 cells in 200 μL of a serum-free medium were added to the apical chamber, and 300 μL of the same medium containing 10 % FBS was placed in the bottom chamber. The plates were incubated for 24 h at 37°C in 5% CO2, and then the cells were fixed in methanol for 15 min and stained with 0.1% crystal violet in PBS for 15 min. The non-migrated cells on the upper side of the filters were scraped gently with cotton-tipped swabs, and the filters were washed with PBS. The cells on the lower face of the membrane were examined and counted under a fluorescent microscope in five randomly selected fields of 100X magnification. Each experiment was repeated at least 3 times.

Invasion assay

To determine cell invasion, pre-coated with Matrigel invasion chambers (Costar, NY, USA; pore size: 8 μm) were used according to the manufacturer’s protocol. 1 × 10e5 resuspended cells in 200-μl serum-free medium per well were transferred to the upper chamber of the Matrigel-coated inserts; 300 μL of the same culture medium containing 10 % FBS was placed in the bottom chamber. The cells were incubated for 24 h at 37 °C in 5% CO2. After incubation, the cells were fixed in methanol for 15 min and stained with 0.1% crystal violet in PBS for 15 min. The non-invaded cells on the upper membrane surface of the filters were scraped gently with cotton-tipped swabs, and the filters were washed with PBS. The cells passed through the filter on the lower face of the membrane were stained and counted under a fluorescent microscope in five randomly selected fields of 100X magnification. Each experiment was repeated at least 3 times.

Colony-forming assay

Cells were seeded in duplicate into a 35-mm plate at a density of 5×103 cells per 1.5-mL methylcellulose-based media (R&D Systems) according to the manufacturer's instructions and cultured for 10 to 14 days at 37℃/5% CO2 in a humidified atmosphere.

Luciferase assay

143b and MG63 cells with OLR1 shRNA plasmids or relevant controls were plated in 24-well plate. Plasmids containing NF-κB promoters were transfected using Lipofectamine 2000. 24 h later, cells were lysed and centrifuged. The supernatants were used for luciferase detection using the Luciferase kit (Sigma, St Louis, MO, USA), according to the manufacturer's protocols.

In vivo tumor xenograft study

Four- to six-week-old female immunodeficient NOD/SCID Balb/c mice were purchased from Beijing HFK Bioscience Co. Ltd. Experiments were carried out in 6- to 16-week-old mice, then maintained in our animal facility under pathogen-free conditions in accordance with the Institutional Guidelines and approval by local authorities.

For the investigation of the metastatic lung tumors, lung metastases were allowed to develop for 8 weeks after 143b cell injection. 143b-shRNA cells (2 × 106 cells resuspended in 0.1 mL of PBS /mouse) or control 143b-NC cells (2 × 106 cells resuspended in 0.1 mL of PBS /mouse) were inoculated subcutaneously into the back space or i.v. injected into the lateral tail vein (10 animals per group and repeated twice). The primary tumor size was measured every week, and the mice were monitored 3 times per week for evidence of morbidity associated with pulmonary metastases. After 8 weeks, the mice were anesthetized with 4% chloral hydrate (0.2 ml/100 g, i.p.) and sacrificed by cervical dislocation. Subsequently, the lungs were harvested, fixed in 10% formalin, embedded in paraffin, and examined using hematoxylin and eosin (H&E) staining for morphology. The tumor volume was measured in 2 perpendicular dimensions (D1, D2) with an electronic digital caliper and calculated using the formula: V = 4/3π[1/4(D1 + D2)]2. To quantify the number of pulmonary metastatic lesions, sequential serial 3-μm-thick sections of whole lungs were obtained. The sections were stained with H&E to identify the metastases by light microscopy. All sections were reviewed and interpreted by 2 senior pathologists (Shumei, Yan and Yong, Li).

**Additional file 1: Figure S1**


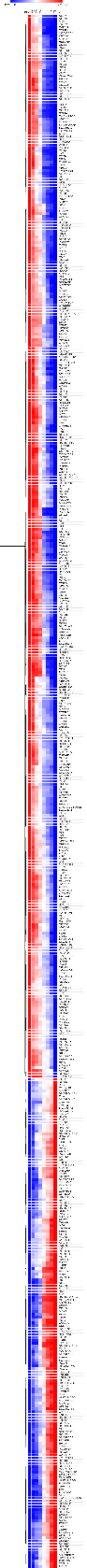


Figure S1. A high-throughput method to screen for activated metastasis-driving genes in osteosarcoma. Heatmap clustering of expression array data obtained from 4 pairs of primary and metastatic tumors tissues.
